# Credence of device acceptability: a statistical method for comparing blood pressure measurement device accuracy across studies

**DOI:** 10.1038/s41371-025-01040-6

**Published:** 2025-07-10

**Authors:** Andrew Lowe, Yang Yu, Tanvi Chandel

**Affiliations:** Institute of Biomedical Technologies, Auckland University of Technology, Auckland, New Zealand

**Keywords:** Diagnosis, Hypertension

## Abstract

Accurate blood pressure measurement (BPM) is critical for managing hypertension, a leading global health concern. While international standards like ISO 81060-2 are applied to ensure commercial BPM device safety and effectiveness, gauging compliance earlier in research and development can be challenging. This study proposes an enhanced statistical evaluation framework that calculates a credence of device acceptability, aligned with international standards, which can be used to assess and compare results of device evaluation experiments having various sample sizes, blood pressure ranges, mean, standard deviation and correlations in error. Applied to ten studies featuring diverse BPM methods, the framework demonstrates its capability to provide insights beyond the face-value application of the performance criteria of international standards. This framework advances BPM technology by providing more appropriate tools to assess device accuracy.

## Introduction

Globally, it has been reported that approximately 12.8% of deaths and 4.4% of disability-adjusted life years are caused by abnormal blood pressure. Moreover, the leading cause of the worldwide burden of disease is high blood pressure (hypertension) [1]. Hypertension management is always a formidable task, from accurate measurement to effective treatment [2]. The American Heart Association classifies blood pressure (BP) and corresponding diagnosis in four categories according to the systolic blood pressure (SBP) and diastolic blood pressure (DBP) levels: normal, elevated, stage 1 hypertension, and stage 2 hypertension [3]. The blood pressure measurement (BPM) is a well-known clinical method to monitor cardiovascular function, and it is also a strong predictor of death and cardiovascular disease [4]. The history of BPM can be traced back to the end of the 18th century [5]. During these decades, many advancements in concept and technology of BPM have allowed us to diagnose abnormal blood pressure and predict cardiovascular pathologies effectively. However, the research on sphygmomanometers does not stop there. New techniques with more portability (e.g., ambulatory devices) and acceptable accuracy have gradually become standardised and gained consensus.

Over the years, several prestigious organizations have developed clinical validation protocols for BPM devices, such as the American National Standards Institute (ANSI)/ the Association for the Advancement of Medical Instrumentation (AAMI) [6], the BHS Protocol by the British Hypertension Society [7], and the ESH International Protocol by the European Society of Hypertension [8]. International regulations have converged on the current International Organization for Standardization “ISO 81060-2:2019 Non-invasive sphygmomanometers. Part 2: Clinical investigation of intermittent automated measurement type”. With recent advances in cuffless (which is technically out of scope for ISO 81060-2) and wearable devices, standards organisations have responded with the IEEE standard for Wearable, Cuffless Blood Pressure Measuring Devices by the Institute of Electrical and Electronics Engineers [9] and ISO 81060-3:2022 Non-invasive sphygmomanometers Part 3: Clinical investigation of continuous automated measurement type [10]. In addition, ISO 81060-7 which will cover cuffless BPM devices is being drafted. Such standards specify consistent approaches throughout the clinical investigation, from validation to statistical analysis [11]. Compliance with these standards is particularly valuable to give confidence that devices to be placed in market have acceptable characteristics and performance for the typical use cases envisioned by the standards.

Taking the widely used ISO 81060-2 as an example [6], this standard applies to all sphygmomanometers intended for diverse patient populations and various usage conditions. It mandates that clinical investigations employ either a noninvasive auscultatory BPM at the upper arm or an invasive BPM as the reference standard. For studies using an auscultatory reference BPM, the protocol requires a minimum of 85 subjects and at least 255 valid paired BP values. Furthermore, the demographic composition must include at least 30% male and 30% female subjects. The standard also prescribes a wide BP distribution: for reference SBP readings, at least 5% should be below 100 mmHg, at least 5% above 160 mmHg, and at least 20% above 140 mmHg; for reference DBP readings, at least 5% should be below 60 mmHg, at least 5% above 100 mmHg, and at least 20% above 85 mmHg. Additionally, the standard specifies requirements for cuff and limb sizes, age distribution, data analysis, and adjustments for special patient populations, ensuring comprehensive and rigorous validation procedures.

Originally, for example in ANSI/AAMI SP10:1992 manufacturers were required to keep the mean error within ± mmHg and the standard deviation under 8 mmHg. These limits were intended to ensure that the error in any single measurement was less than a tolerable error (decided by the writers of the standard) with some probability, $\hat{p}$ (also decided by the writers of the standard). However, these fixed limits did not account for the relationship between the mean and standard deviation of errors. For the same standard deviation, the probability of tolerable error varies depending on whether the mean error is 0 mmHg or 5 mmHg. To address this, later standards including SP10:2002 and the current ISO 81060-2 introduced criteria that adjusts the upper standard deviation limit based on different mean errors, ensuring 85% of errors fall within an acceptable range ($\hat{p}$ = 0.85) [6]. For example, with a mean error of 0 mmHg, the standard deviation must be ≤ 6.95 mmHg for the device to be accepted.

To date, much reported research does not adhere to the ISO standards in these recently published continuous BP standards. This is particularly the case for research groups exploring new BPM technologies at early stages, due to the cost associated with complying with the stipulations of the standards. This situation highlights the gap between the desire to adopt new, continuous BP monitoring technology and the paucity of evidence from research studies in full compliance with international validation standards. The most direct way to address this gap is to conduct full-scale validation. However, this is time consuming, costly, and may even raise ethical questions about the demands placed on participants in studies that are needlessly overpowered to confirm or refute whether devices can meet performance expectations.

International standards such as ISO 81060-2 and the AAMI/ANSI SP10 require a minimum sample size of *N* ≥ 85 participants. However, given practical constraints, many research studies utilize smaller sample sizes. In previous research [12], to evaluate the performance of different BPM methods in studies utilizing smaller sample sizes, our group developed statistical methodologies consistent with ISO 81060-2, which explored changes in the acceptance region (combinations of mean and standard deviation of error) for varying sample sizes. The original derivation of the criteria used in international standards, as described in AAMI/ANSI SP10:2002 + A1:2003 + A2:2006 Annex F, relied on Gaussian approximations, using Taylor expansions around the mean and standard deviation to provide confidence limits for *p*, the true probability of tolerable error. However, the method described in the standard produces a biased standard error, dependent only on the sample size *N*, whereas our previous work proposed a sampling distribution approach, where the standard error depends not only on *N* but also on the mean error and standard deviation of the errors, yielding a more accurate approximation than the framework provided by the standard, and also provided a methodology to evaluate the probability of acceptance (*P*_*A*_), which is the probability of meeting the ISO 81060-2 accuracy criteria. This approach facilitated a comparative analysis of accuracy across different studies with varying sample sizes. The accuracy evaluation was based on *P*_*A*_ values, where *P*_*A*_ ≥ 95% is the threshold for acceptability as per the standard.

In the current study, we aim to extend this proposed framework by incorporating BP ranges, in addition to sample size, mean error, and standard deviation, to evaluate the credence of device acceptability. This expansion allows for a more comprehensive evaluation of BP measurement device accuracy across different clinical settings, because many studies with smaller sample sizes also measure from a narrower range of reference BPs than required by the standards. Our objective is to refine the acceptance criteria for BP devices, ensuring that they account for varying BP ranges, which is crucial to device reliability and patient outcomes. By expanding the framework, we aim to better guide device evaluation across diverse clinical scenarios and improve application of BPM device criteria.

The following section outlines the methodology for evaluating the credence of device acceptability. A detailed mathematical treatment is provided in the Appendix. The results section will present a comparative analysis of the credence of device acceptability for previous studies utilizing different BPM techniques, incorporating insights from each technique. In the discussion section, the limitations of the current approach will be addressed, along with recommendations for future research directions.

## Method

### Overview of statistical method

Standards universally define the error in a measurement as the difference between the reference BP reading (which may itself be an average of readings) and the measurement from the device-under-test (DUT). The maximum error deemed ‘acceptable’ (which approximates ‘remaining clinically useful’), also known as tolerable error, is denoted by Δ. Standards require a level of confidence, *C*_*pmin*_, that a DUT is acceptable, defined as the probability that the true proportion of tolerable errors is no less than a specified threshold, *p*_*test*_. Any single validation experiment will provide an estimate of the true proportion of tolerable errors within some confidence limits, which will depend on the number of paired reference and DUT measurement samples, *n*, collected during the experiment. More samples will result in a narrower confidence interval. Standards therefore also stipulate a sample size designed to ensure a minimum level of confidence that the acceptance criteria are met. Implicit in this specification is the assumption that the experimental measurements come from a process that is like the one assumed in deriving the accuracy criteria. For example, the phenotypes of participants should match what is assumed by the standard, including similar ranges of blood pressure.

**Fig. 1:**
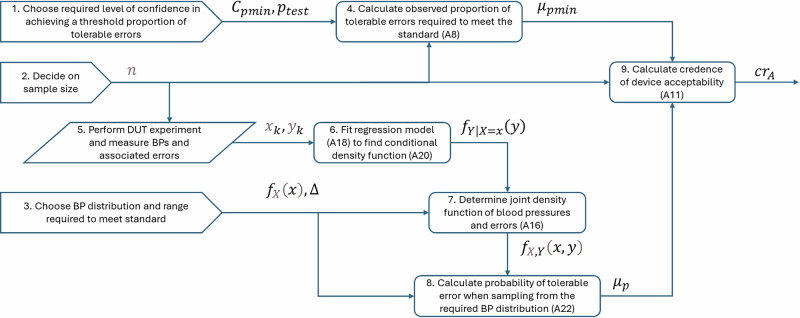
Flow chart showing the steps in calculating credence of device acceptability. Symbols and bracketed numbers correspond to equations in the appendix.

We wish to calculate a score that reflects the probability that a DUT will meet standardised accuracy criteria. This score should account for the fact that an available experiment will have different sample size and different blood pressure range than stipulated by the standardised test. We call this score the “credence of device acceptability”, *cr*_*A*_, and define it as the probability that the proportion of tolerable errors, as evidenced by an experiment with the DUT, is no less than some threshold, *μ*_*pmin*_, that corresponds to the proportion of tolerable errors that would be expected from a standards-compliant device if it were to be subjected to the same experimental design as used for the DUT. In the following paragraphs we present an explanation of the calculation of *cr*_*A*_, with reference to the flow chart in Fig. 1. The full derivation is given in the appendix.

Parameters for acceptable performance must be chosen. These parameters, which have been described above, are *C*_*pmin*_, *p*_*test*_ (block 1) and Δ (block 3). The distribution of blood pressures required by the standard should also be known, and in our derivation is represented by the random variable, *X*, and its probability density function, *f*_*X*_ (block 3). The last remaining parameter to choose is the number of samples, *n*, in the experiment with the DUT (block 2). From this information, using Eq. (A8), we can determine the acceptance threshold described above, *μ*_*pmin*_ (block 4).

The DUT validation experiment will yield *n* paired BP and BP error values, *x*_*k*_ and *y*_*k*_, respectively (block 5). Fitting a linear regression model (block 6) to these data gives the probability density function of the error conditional on the measured blood pressure, *f*_*Y*|*X*=*x*_(y). Combining this with previously chosen *f*_*X*_ allows calculation (block 7) of the joint density function *f*_*X,Y*_(*x*,*y*). The joint density function represents the likelihood of any combination of measured BP and measurement error, when measuring using the DUT and sampling from a population with BP distribution required by the standard. From this, we can calculate the probability *μ*_*p*_ by integrating between limits of tolerable error (-Δ to Δ) for all BP (block 8). To finish, credence of device acceptability can be calculated (block 9).

### Application

To support readers and researchers who wish to apply the proposed mathematical method for estimating the expected performance of a BPM device, we have developed a user-friendly online calculator [13] powered by Wolfram Cloud. This tool allows users to easily compute the credence metric and results plots by inputting the mean and SD of the BP distribution and the measured error characteristics.

To align with the requirements and statistical assumptions underpinning current standards, the following parameter values have been used:

$${C}_{{pmin}}=0.95;{p}_{{test}}=0.78;\Delta =10{mmHg}$$

The BP distribution presumed by the standard is represented by normal distributions (i.e. *X*~*N*) with specifying mean ± standard deviations: 130 ± 20 mmHg for systolic BP, and 80 ± 13 mmHg for diastolic BP, respectively.

The rationale for these values is as follows: In ANSI/AAMI SP10:2002 Annex F, 10 mmHg is defined as the tolerable error, a device is considered acceptable if its estimated probability of a tolerable error is at least 85%, but with a sample size of 85, there is a 95% chance that the estimated probability of a tolerable error will be at least 78% (or, as described in SP10, a 90% chance that the difference is no more than 7%). ISO 81060-2:2019 + A1:2020 section 5.2.4.1.2 retains the same mean-error-dependent acceptance criteria as SP10, and also specifies blood pressure distributions for auscultatory reference measurements (section 5.1.5) that are met by the parameter values for *X* given above, assuming blood pressures are normally distributed. These values ensure that at least 5% of SBP readings fall below 100 mmHg, at least 5% above 160 mmHg, and at least 20% above 140 mmHg, while similarly meeting the thresholds for DBP: at least 5% below 60 mmHg, 5% above 100 mmHg and 20% above 85 mmHg.

To illustrate the proposed evaluation algorithm, we did a brief literature review to identify several previous studies that employed various BPM methods. These studies were selected to ensure their relevance to this research:

1. The studies had to focus on BPM methods, ensuring alignment with the scope of our evaluation framework.
2. The studies needed to describe clear validation protocols, including sufficient statistical parameters to enable algorithmic calculations, including sample size, ranges of SBP and DBP, mean error and its standard deviation.
3. The BP data used in these studies had to originate from measurements obtained directly from participants, rather than relying on online databases or simulated datasets.

## Results

**Fig. 2:**
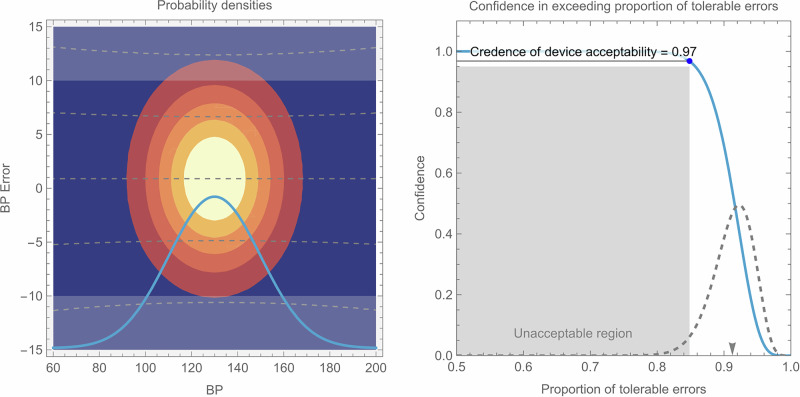
Example of BPM [14] with high credence of device acceptability for SBP. Reported (mean ± SD) systolic BPs were 131.4 ± 21.35 mmHg and BP errors were 0.89 ± 5.65 mmHg with 85 samples.

**Fig. 3:**
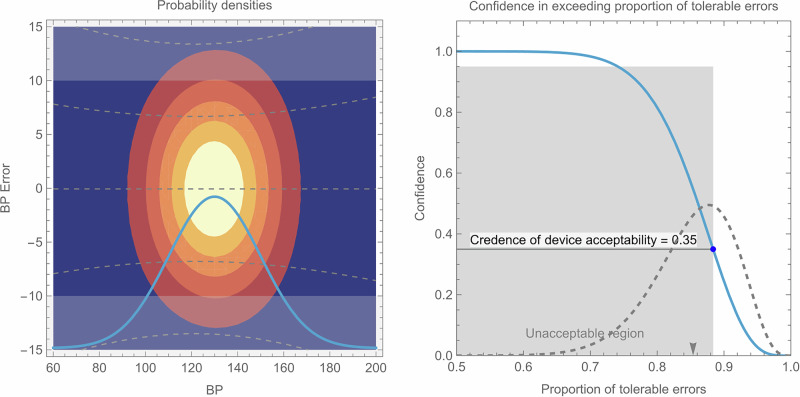
Example of BPM [15] with low credence of device acceptability for SBP. Reported (mean ± SD) systolic BPs were 121.4 ± 17.6 mmHg and BP errors were −0.06 ± 6.63 mmHg with 33 samples.

Ten previous studies reporting device inaccuracies are summarized in Table 1. The inaccuracies presented in the table represent the error (Mean ± SD) associated with each BPM method, measured in mmHg. Although our technique can account for systematic errors in BPM (that is, correlation between error and BP), this information is not generally reported and therefore omitted from the analysis. The presented examples encompass a wide range of BPM techniques, including the widely used cuff-based oscillometric method, the finger volume-clamp method, pulse transit time (PTT)-based approaches, and machine learning (ML)-driven pulse wave analysis methods. For instance, a recent validation study evaluated the accuracy of a newly developed wrist-type automatic BPM in accordance with the ISO 81060-2 standard, obtaining a credence of device acceptability exceeding 99% for both SBP and DBP (as shown in Fig. 2) [14]. Conversely, an early investigation into cuffless BPM using the PTT-based method indicated a lower credence of device acceptability for SBP, attributed to a limited participant number and restricted BP range, as illustrated in Fig. 3 [15].

**Table 1:** Comparison statistics of the previous clinical studies.

Studies	Methods	Sample Size	BP Range (mmHg) (mean ± SD)		Reported Device Inaccuracy (mean ± SD)		Evaluation Results			
			SBP	DBP	SBP	DBP	Credence of device acceptability		Probability of a tolerable error for BPs in the target population	
							SBP	DBP	SBP	DBP
[17]	Oscillometric method with PTT	85	130.42 ± 17.56	97.7 ± 13.15	−0.01 ± 7.98	0.01 ± 5.39	6.50%	99.20%	78.40%	93.00%
[14]	Oscillometric method on wrist	85	131.4 ± 21.35	84.3 ± 15.7	0.89 ± 5.65	−1.44 ± 5.61	96.80%	94.10%	91.30%	90.30%
[18]	Oscillometric method on wrist	33	140.1 ± 23.7	87.1 ± 16.2	−0.7 ± 6.9	−1 ± 5.1	25.20%	87.60%	83.70%	93.40%
[19]	Smart phone-based finger oscillometric method	31	107 ± 8.5	74.5 ± 7.25	3.3 ± 8.8	−5.6 ± 7.7	<0.1%	<0.1%	61.00%	60.50%
[20]	Finapres	102	127.98 ± 20.34	78.5 ± 12.03	−1.83 ± 6.83	0.88 ± 7.49	33.40%	18.50%	82.50%	80.80%
[21]	PTT with ML algorithms	3077	123.6 ± 21.2	83.4 ± 12.1	2.31 ± 9.57	1.33 ± 6.43	<0.1%	99.90%	67.60%	86.40%
[22]	PTT(PAT) using ECG and PPG	22	128.5 ± 8.2	76 ± 7.7	0.01 ± 9.69	0.04 ± 9.68	0.10%	0.20%	64.10%	66.10%
[15]	PTT(PAT) using ECG and PPG	33	121.4 ± 17.6	76.7 ± 10.5	−0.06 ± 6.63	−0.25 ± 5.63	35.00%	75.20%	85.40%	91.20%
[23]	BIM on the finger with ML algorithms	10	151 ± 20.67	82 ± 13.33	0.11 ± 5.27	0.11 ± 3.87	46.00%	90.20%	90.20%	98.00%
[24]	PPG on the finger with ML algorithms	89	127.5 ± 15.83	82.5 ± 13.17	0.16 ± 5.9	−0.07 ± 4.68	95.50%	99.90%	90.50%	96.50%

*SBP* systolic blood pressure, *DBP* diastolic blood pressure, *ECG* electrocardiogram, *PPG* photoplethysmography, *BIM* bioimpedance measurement, *ML* machine learning, *PAT* pulse arrival time, *PTT* pulse transit time, *SD* standard deviation.

In each of these figures, the left-hand plot shows contours of the joint density function *f*_*X,Y*_(*x*,*y*). This highlights the area of most likely combinations of BP and BP error. The horizontal light-shaded bands at the top and bottom of the chart show the regions of error outside of what is considered tolerable. The superimposed bell-curve (solid line, arbitrary vertical scaling) is the probability density function, *f*_*X*_, of the required BP distribution. The dashed lines show the mean prediction and 68 and 95% individual prediction limits for BP errors, derived from the experiment’s regression model. The 95% individual prediction limits correspond closely with Bland-Altman “limits of agreement” but also account for the range of sampled BPs.

The right-hand plot shows, given the experimental data and parameter choices made, how confidence in the DUT exceeding a proportion of tolerable errors changes with the value of proportion of tolerable errors (solid line). The gray zone represents the unacceptable region bounded by *μ*_*pmin*_ on the right and *C*_*pmin*_ on top, so that if the solid line intersects the gray zone, the DUT is not expected to meet the requirements of the standard. The credence of device acceptability score is the confidence associated with *μ*_*pmin*_ and is shown as a point on the solid line. Also on the right-hand plot, the experimentally determined proportion of tolerable error, *μ*_*p*_, is shown with an arrow-head on the horizontal axis, and the modelled distribution of *P* (i.e. the anticipated distribution of *μ*_*p*_ from multiple experiments) is shown using a dashed line (arbitrary vertical scaling). The arrow-head is the mean of the distribution, and the width of the distribution is an indication of the standard error of the mean.

Compared to Fig. 2, Fig. 3 shows a greater spread of measurement errors, even though the mean error is closer to zero. Furthermore, the smaller sample size and smaller range of measured BPs contributes to more divergent individual prediction bands in Fig. 3. Together, these significantly increase the variance of *P* (seen as the width of the dashed line in the right-hand plot) which reduces the credence of device acceptability, even though, at face value, both studies report error statistics that meet the ISO 81060-2 criteria.

It is important to emphasize that this re-evaluation and critique do not undermine the significant contributions of these studies to the field; instead, our research can be used to underscore areas for improvement that can advance the accuracy and reliability of BPM technologies, and help to synthesise the findings of the various reported studies.

## Discussion

Accurate BPM is critical for the effective management of cardiovascular health, as it serves as a cornerstone for diagnosing hypertension and assessing cardiovascular risk. Inaccurate BPM may lead to misdiagnosis and inappropriate treatment [16]. International standards such as ISO 81060-2 play a vital role in ensuring that BPM devices meet safety and effectiveness criteria, providing essential guidance for consumers, manufacturers, and regulators. The pass/fail criteria outlined in these standards serve as a benchmark for defining acceptable levels of error in BPM, thereby establishing trust in device performance. Thus, adhering strictly to international standards is the most effective and reliable approach to ensuring the accuracy of BPM devices.

Developing and validating novel BPM techniques often faces significant logistical and methodological challenges. Full-scale validation studies, though ideal, are frequently impractical during the development phase due to constraints such as limited time, budgetary restrictions, and the complexities of obtaining ethical approval. These challenges can lead to insufficient sample sizes, unrepresentative BP distributions, and other recruiting biases which may compromise the generalizability of the findings. For instance, if a new BPM technique is tested exclusively on participants with normal BP, its efficacy in measuring and tracking elevated BP in hypertensive patients remains uncertain. Moreover, many published studies lack comprehensive statistical information, making it difficult to objectively compare results across different studies or to assess the robustness of a given technique. Asking researchers to repeat their experiments to address such gaps is neither feasible nor efficient.

In this study, we proposed an algorithm to evaluate the credence of device acceptability, which provides a valuable tool for assessing the accuracy and reliability of BPM devices during their development phase and comparing the performance of various techniques. By introducing a statistical framework that aligns with ISO 81060-2 standard, this method enables researchers to evaluate device performance prior to full-scale validation, thus facilitating iterative design improvements and minimizing resource expenditure. For instance, by allowing for early identification of limitations in novel BPM techniques, such as new cuffless BPM methods, our evaluation method can help guide design enhancements that could improve their clinical utility.

As demonstrated in Table 1, our proposed method effectively evaluates and compares the relative performance of different BPM techniques across studies with varying sample sizes, BP distributions, and reported inaccuracies. For studies employing similar measurement techniques, a lower credence of device acceptability was observed when participant numbers were smaller, or BP distributions were narrow and/or not representative of the general population, highlighting the critical impact of sampling on the evaluation outcomes. Notably, the emerging cuffless techniques, such as PTT-based approaches, exhibited significantly lower credence of device acceptability. This finding underscores the need for continued research and development to enhance their accuracy and reliability, ensuring they can achieve clinical-grade performance in real-world applications.

During our short literature review, we observed that many previous studies evaluating new BPM techniques or algorithms continue to rely on the simplistic criteria of “mean error less than 5 mmHg and standard deviation less than 8 mmHg” to claim adherence to ISO standards. However, we have demonstrated that these pronouncements do not account for critical factors such as small participant sample sizes and limited BP ranges and risk presenting an overly optimistic view of device performance, potentially misleading uninformed readers and obscuring the true performance of the proposed methods.

Despite its utility, our approach has remaining limitations. The current framework assumes a normal distribution of BP and measured errors, which may not fully capture the variability in device performance across different BPM technologies or patient populations. Future research could explore alternative statistical models, such as t-distributions, to assess whether they provide significantly different evaluations under non-normal conditions. Additionally, our analysis does not account for many other sources of potential bias in pre-existing validation data. International standards already recognise some such biases, including arm circumference, gender, age and special patient populations, but there are likely to be others that affect the novel BPM methodologies published in recent research. Future developments of our framework could address such issues to refine the assessment of credence of device acceptability, but this would require statistical information about their association with measurement errors that is not readily available. Another limitation is that we do not account for different cross-validation schemes potentially employed during device development and testing. Ideally, devices are tested on participants, none of whom contributed data during device development. However, papers do not typically report cross-validation methods in sufficient detail to account for departures from this ideal. Conversely, examining the variability in the calculated credence over repeated experiments of the same design could be a way uncover sources of variability due to non-obvious methodological effects or, at least, evaluate the real-world repeatability of the credence metric itself. For now, we hope that our research serves to increase awareness about the implications of deviations from standardised validation protocols. It does not preclude the need to adhere to the full standard for regulatory clearance and to support clinical acceptance.

## Conclusion

Our research provides a statistical analysis method that provides a practical and scientifically rigorous solution to evaluate the credence of BP device acceptability. Our method enables researchers to gain more rigorous insight into device performance, and more objectively compare reported studies with varying participant numbers, BP distributions, and correlation between BP errors and BP (i.e. systematic errors). By applying this analysis, researchers, device developers and clinicians can better synthesise conclusions from disparate studies, identify areas for improvement in emerging techniques, such as cuffless and wearable BPM devices, draw more valid conclusions from and better gauge clinical relevance of smaller and lower-cost clinical evaluations. Ultimately, the proposed framework advances the field by offering an objective, flexible tool to support device development and validation, supporting the creation of more reliable BPM technologies to benefit diverse patient populations.

### Appendix: statistical derivation

#### Credence threshold

For a given device validation experiment, we will have a sample size, *n*, and proportion of acceptable errors *μ*_*p*_. The standard error of this sample proportion is

A1 $${\sigma }_{p}=\sqrt{\frac{\left(1-{\mu }_{p}\right){\mu }_{p}}{n}}$$

We assume the random variable, *P*, representing the proportion of acceptable errors from repeated experiments can be described by

A2 $$P \sim {{{\rm{Beta}}}}\left[\alpha ,\beta \right]$$

Parameters *α* and *β* are chosen such that the mean and standard error of the mean are equal to the experimentally observed proportion and standard error:

A3 $$\alpha =\frac{{\mu }_{p}^{2}-{\mu }_{p}^{3}-{\mu }_{p}{\sigma }_{p}^{2}}{{\sigma }_{p}^{2}},\beta =\frac{(-1+{\mu }_{p})(-{\mu }_{p}+{\mu }_{p}^{2}+{\sigma }_{p}^{2})}{{\sigma }_{p}^{2}}$$

The cumulative distribution function of *P* is

A4 $${{{{\mathscr{F}}}}}_{P}[p]=\Pr [P \; < \;p]={{{{\rm{I}}}}}_{p}[\alpha ,\beta ]$$

where Pr[▪] denotes a probability, *F*_*P*_[▪] is the cumulative distribution function for random variable *P*, and I_*p*_[*α*, *β*] is the regularised beta function.

We define the confidence that a device is acceptable in terms of the probability that the true proportion of tolerable errors is greater than a test proportion, *p*:

A5 $${C}_{p}=\Pr [P\ge p]=1-\Pr [P \, < \, p]$$

A6 $${C}_{p}=1-{{{{\rm{I}}}}}_{p}[\alpha ,\beta ]$$

Substituting and simplifying using previous Eqs. (A3) and (A1) show that this is a relationship between *C*_*p*_, *p*, *μ*_*p*_ and *n*:

A7 $${C}_{p}=1-{{{{\rm{I}}}}}_{p}[\left(n-1\right){\mu }_{p},\left(n-1\right)({1-\mu }_{p})]$$

Standards specify, for device acceptability, the required level of confidence, *C*_*pmin*_, calculated at a test proportion, *p*_*test*_, of tolerable errors. This relationship therefore determines the observed proportion of tolerable errors required to achieve device acceptability, *μ*_*pmin*_.

A8 $${C}_{{pmin}}=1-{{{{\rm{I}}}}}_{{p}_{{test}}}[\left(n-1\right){\mu }_{{pmin}},\left(n-1\right)({1-\mu }_{{pmin}})]$$

**Fig. 4:**
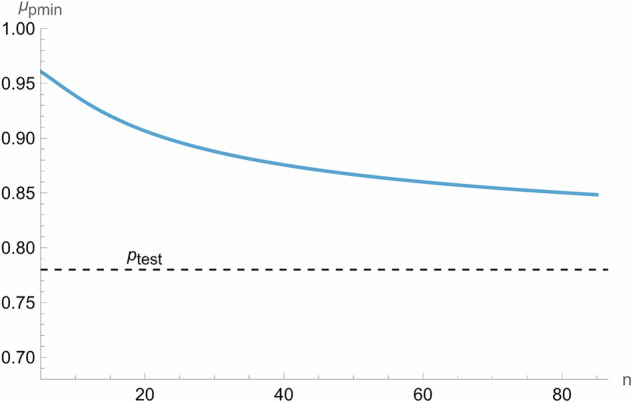
The relationship between acceptance threshold and sample size (P_test_: Test Proportion, μ_pim_: Minimum Observed Proportion of Tolerable Errors, n: sample size).

We can solve this numerically for *μ*_*pmin*_ for chosen values of the other parameters. For example, choosing *p*_*test*_ = 0.78 and *C*_*pmin*_ = 0.95, the relationship between sample size, *n*, and *μ*_*pmin*_ is shown below (Fig. 4):

We define credence of device acceptability, *cr*_*A*_, as the probability that the true proportion of tolerable errors, as evidenced by an experiment with the device under test (DUT) is no less than *μ*_*pmin*_.

A9 $${{cr}}_{A}\triangleq \Pr [P\ge {\mu }_{{pmin}}]=1-\Pr [P \, < \, {\mu }_{{pmin}}]$$

Substituting using Eqs. (A5), (A7) and then (A9) above gives

A10 $$\Pr \left[P \, < \, {\mu }_{{pmin}}\right]={{{{\rm{I}}}}}_{{\mu }_{{pmin}}}[\left(n-1\right){\mu }_{p},\left(n-1\right)({1-\mu }_{p})]$$

A11 $${{cr}}_{A}=1-{{{{\rm{I}}}}}_{{\mu }_{{pmin}}}[\left(n-1\right){\mu }_{p},\left(n-1\right)({1-\mu }_{p})]$$

#### Experimentally determined probability of tolerable error

In Eq. (A11) above, *μ*_*p*_ represents the proportion of tolerable errors found by an experiment that meets criteria for the distribution of measured blood pressures stipulated by the standard. This estimate is conditional on the distribution of blood pressures sampled:

A12 $${\mu }_{p}=\Pr \left[-\Delta \, < \, Y \, < \, \Delta |-{{\infty }} \, < \, X < {{\infty }}\right]$$

where *X* is a random variable representing measured blood pressures with the stipulated distribution (i.e. a distribution consistent with the ranges of measured blood pressure required by the applicable validation standard), and *Y* is a random variable representing corresponding experimental measurement errors.

The probability (A12) can be calculated from joint and marginal probabilities as follows:

A13 $${\mu }_{p}=\frac{\Pr [-\varDelta \, < \, Y < \, \varDelta ,-{{\infty }} \, < \, X \, < \, {{\infty }}]}{\Pr [-{{\infty }} \, < \, X < {{\infty }}]}$$

The right-hand-side probabilities can be found by integrating their respective probability density functions, ${{{\mathcal{f}}}}$

A14 $$\Pr [-\varDelta \, < \, Y \, < \, \varDelta ,-{{\infty }} \, < \, X \, < \, {{\infty }}]={\int }_{-{{\infty }}}^{{{\infty }}}{\int }_{-\varDelta }^{\varDelta }{{{{\mathcal{f}}}}}_{X,Y}[x,y]{{{\rm{d}}}}y\,{{{\rm{d}}}}x$$

A15 $$\Pr [-{{\infty }} \, < \, X \, < \, {{\infty }}]={\int }_{-{{\infty }}}^{{{\infty }}}{{{{\mathcal{f}}}}}_{X}[x]{{{\rm{d}}}}x=1$$

To find the joint density function ${{{{\mathcal{f}}}}}_{X,Y}$ we use the identity

A16 $${{{{\mathcal{f}}}}}_{X,Y}[x,y]={{{{\mathcal{f}}}}}_{X}[x]{{{{\mathcal{f}}}}}_{Y|X=x}[y]$$

For the conditional density function, ${{{{\mathcal{f}}}}}_{{Y|X}=x}$ we presume that sampled blood pressure errors, *y*, are normally distributed about a linear regression line, so that we may write

A17 $$\left(Y|X=x\right){{{\mathscr{ \sim }}}}{{{\mathscr{N}}}}[\,\hat{y},{{{{\rm{SE}}}}[x]}^{2}]$$

A18 $$\hat{y}=\bar{y}+{\beta }_{1}\left(x-\bar{x}\right)$$

where $\bar{x}$ and $\bar{y}$ are the sample means of blood pressure and measurement error, and *β*_1_ is the gradient of the regression line. In this notation, the normal distribution is parameterised by mean and variance.

Under these assumptions, the standard error for an individual prediction as a function of measured blood pressure, *S*E[*x*], is given by

A19 $${{{\rm{SE}}}}[x]=\sqrt{\left({\bar{y}}^{2}+{s}_{y}^{2}\right)\left(1+\frac{1}{n}+\frac{{\left(x-\bar{x}\right)}^{2}}{\left(n-1\right){s}_{x}^{2}}\right)}$$

where *s*_*x*_ and *s*_*y*_ are the sample standard deviations of blood pressure and measurement error, respectively.

The equations above fully determine the conditional density functions. The probability density and cumulative distribution functions, written in full for reference, are:

A20 $${{{{\mathcal{f}}}}}_{Y|X=x}[y]=\frac{{{{{\boldsymbol{e}}}}}^{-\frac{{(y-\bar{y}-\left(x-\bar{x}\right){\beta }_{1})}^{2}}{2({\bar{y}}^{2}+{s}_{y}^{2})(1+\frac{1}{n}+\frac{{(x-\bar{x})}^{2}}{(n-1){s}_{x}^{2}})}}}{\sqrt{2\pi }\sqrt{\left({\bar{y}}^{2}+{s}_{y}^{2}\right)\left(1+\frac{1}{n}+\frac{{\left(x-\bar{x}\right)}^{2}}{\left(n-1\right){s}_{x}^{2}}\right)}}$$

A21 $${{{{\mathscr{F}}}}}_{Y|X=x}\left[y\right]=\frac{1}{2}{{{\rm{erfc}}}}\left[\frac{\bar{y}-y+{\beta }_{1}\left(x-\bar{x}\right)}{ {\sqrt {2}}\sqrt{{\left({\bar{y}}^{2}+{s}_{y}^{2}\right)\left(1+\frac{1}{n}+\frac{{\left(x-\bar{x}\right)}^{2}}{\left(n-1\right){s}_{x}^{2}}\right)}}}\right]$$

where erfc[▪] is the complementary error function.

*μ*_*p*_ can then be calculated as follows:

A22 $${\mu }_{p}={\int }_{-{{\infty }}}^{{{\infty }}}{\int }_{-\varDelta }^{\varDelta }{{{{\mathcal{f}}}}}_{X,Y}[x,y]{{{\rm{d}}}}y\,{{{\rm{d}}}}x$$

with which we have fully defined *cr*_*A*_.

## Summary table

### What is known about the topic

- International standards like ISO 81060-2 ensure blood pressure measurement (BPM) device safety and effectiveness but are designed primarily for large-scale validation studies.
- Early-stage evaluation of BPM devices in research and development is challenging due to constraints like limited sample sizes and narrow BP ranges.
- Current BPM evaluation often uses simplistic criteria, such as mean error <5 mmHg and standard deviation <8 mmHg, which may not fully account for variability and systematic biases.

### What this study adds

- Proposes a statistical framework for calculating the credence of device acceptability, accommodating varying sample sizes, BP ranges, and reported errors.
- Demonstrates the framework’s ability to provide an objective comparison of BPM device accuracy across different studies, even with limited resources.
- Highlights critical factors impacting BPM evaluation, offering a more robust methodology aligned with international standards.

## Data Availability

The data that support the findings of this study are available from the corresponding author upon reasonable request.
